# Hungary's Current Climate Conditions Converge With the North‐Mediterranean of the 1980s: A Case Study in Mediterranean Ant Species New to Hungary

**DOI:** 10.1002/ece3.73096

**Published:** 2026-02-17

**Authors:** Sándor Csősz, Miklós Laczi, Gábor Herczeg, Ferenc Báthori

**Affiliations:** ^1^ Department of Systematic Zoology and Ecology, Institute of Biology ELTE Eötvös Loránd University Budapest Hungary; ^2^ HUN‐REN‐ELTE‐MTM Integrative Ecology Research Group Budapest Hungary

**Keywords:** Carpathian Basin, climate change, Formicidae, global warming

## Abstract

Climate change reshapes our environment, affects species distributions, and threatens biodiversity. Our warming climate allows species to broaden their ranges naturally by expanding or shifting their distribution areas towards available, previously unsuitable areas, or anthropogenically by establishing breeding populations followed by active dispersion after intentional or unintentional human introduction. This effect, coupled with the ongoing intensification of human transport of goods, accelerates the rate of biological invasions considerably. Ants have a remarkable ability to disperse and become invasive, resulting in profound ecological and economic damage. Most relevant studies in the Carpathian Basin (Central Europe) focus solely on identifying new non‐native species, typically without exploring the relationship between climate change and species invasions. Here, we studied how climate change might aid Mediterranean ants' invasion of the Carpathian Basin. First, we monitored non‐native Mediterranean ant species in Hungary (occupying most of the Carpathian Basin) and recorded four of such species. Then, we compared the historical Mediterranean climate (1980s) with the current Hungarian climate of the four species' habitats to assess whether Hungarian conditions have converged with those Mediterranean conditions from which these species originate. We found that the climate of both areas changed significantly during the last 40 years by becoming hotter and drier. This change resulted in the disappearance of the climatic differences that previously existed between the 1980s Mediterranean region and 1980s Hungary (higher radiation and minimum temperatures in the former). When comparing 1980s Mediterranean climate to current Hungarian climate, these differences have largely vanished. Our results demonstrated that climate change could make Hungarian habitats climatically suitable for Mediterranean species in 40 years, implying significant risks of invasions currently and in the near future.

## Graphical abstract

Climate change reshapes our environment, affects species distributions, and threatens biodiversity. We show that historical climate of certain Mediterranean ant species' native areas are similar to the contemporary climate of the same ant species' non‐native Hungarian areas/occurrences. Our results demonstrate that climate change could make Hungarian habitats climatically suitable for Mediterranean species in 40 years, implying significant risks of invasions currently and in the near future.

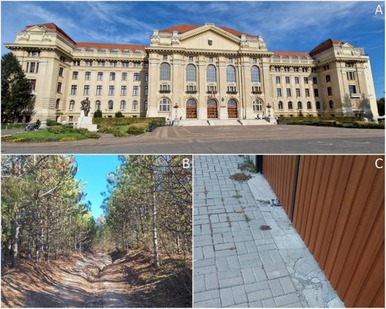

## 1 Introduction

The introduction of non‐native species has increased significantly, especially since the 1970s, making invasive species a key contributor to biodiversity loss and economic challenges (Chiu et al. 2023). According to forecasts, the number of introduced non‐native species may increase by 36% from 2005 to 2050 (Seebens et al. 2017). The effects of invasive species on new environments can range from minimal to severe ecological and economic consequences (Williams et al. 2010; Eichhorn et al. 2011; Martin‐Albarracin et al. 2015; Tercel et al. 2023). Current studies highlight the urgency of more comprehensive research on biodiversity (Hoveka et al. 2022; Briscoe et al. 2023; Wernberg et al. 2024) in the era of climate change. This is also true for understanding biological invasions and their rapid increase, where the spread and establishment of non‐native species in new environments may come about through complex interactions between growing trade networks (Hulme 2009; Haubrock et al. 2022) and global climate change (Pauchard et al. 2016; Finch et al. 2021).

The emergence and establishment of an introduced species can hinge on two critical factors: dispersal ability and the suitability of the new environment, including climatic conditions. Addressing these aspects is essential for protecting ecosystems from the harmful impacts of introduced species. Dispersal ability (i.e., the ability that allows species to reach new areas), can be a natural phenomenon via passive dispersal of propagule or passive (e.g., anemochory, zoochory) and active dispersal of individuals (Cousens et al. 2008; Clobert 2012). Passive dispersal can be human mediated (anthropochory), through accidental or deliberate transport (Bilton et al. 2001; Iluz 2010; Sádlo et al. 2018; Gippet et al. 2019). Numerous studies have shown that the increased transport of goods and people significantly affects species distribution: human activities allow species to travel greater distances more quickly than ever before (Banks et al. 2015; Gippet et al. 2019). The growing number of human‐facilitated introductions coupled with potentially increasing areas suitable for establishment (Hufbauer et al. 2012) due to rapid climate change (Schlaepfer and Lawler 2023), on both local and regional scales, present a dangerous mixture. As climate conditions evolve, many species are increasingly able to colonize areas that were once unsuitable or inaccessible, as documented by a growing number of studies on non‐native organisms (Hsiang et al. 2017; Borrelli et al. 2020; Kalkuhl and Wenz 2020; Mormul et al. 2022).

Climate change is known to dramatically impact the distribution of numerous species, both now and in the future (Muluneh 2021; Parr and Bishop 2022; Harvey et al. 2023). This phenomenon not only facilitates the establishment of viable invasive populations but also raises critical concerns about native biodiversity. However, in Hungary (Central Europe), comprehensive analyses that examine the roles of climate change and species invasions are relatively sparse. Several studies tend to focus on merely documenting new sightings or compiling lists of non‐native species, rather than delving into the underlying causes (László et al. 2023; Tóth et al. 2023; Teski et al. 2024). Research on the transformation of natural and urban habitats in the Carpathian Basin due to climate change has increased in recent years (Szabó et al. 2016; Jánosi et al. 2023). The rise of non‐native species in the area (Weiperth et al. 2020; Csiky et al. 2023; László et al. 2023) emphasizes the need for effective prevention and monitoring strategies, along with understanding the environmental factors aiding their establishment.

Therefore, our current research seeks to understand how regional climatic changes affect the distribution of introduced non‐native ant species. We specifically seek to determine whether the significant increase in ant invasions in Hungary can be linked to changing climate. To this end, we aimed to explore the extent to which Hungary's current climate has converged with the Mediterranean climate of the 1980s—the period and region from which these ant species originated (IPCC 2023). Specifically, we investigated whether the climatic barriers that once prevented these Mediterranean species from establishing in Hungary have diminished or disappeared due to regional warming, allowing them to establish thriving populations in Hungary through potential human‐assisted migration.

To address this important question, we selected ants for this purpose because of their remarkable attributes: (i) ants play a crucial role in maintaining healthy ecosystems. (ii) invasive species of ants pose a significant threat, resulting in severe ecological and economic consequences (Griffiths et al. 2018; Wills and Landis 2018), (iii) ants are considered as some of the most devastating invasive species on Earth (Gutrich et al. 2007; O'Dowd et al. 2003; Nagy et al. 2009), (iv) most ants have good dispersal potential, as their winged females can cover relatively large distances (Bertelsmeier et al. 2015; Helms 2018; Vepsäläinen and Pisarski 1982). Further, there is evidence that they can also spread through involuntary human mediation (Hakala et al. 2019; Aulus‐Giacosa et al. 2024). For instance, it is known that small colonies or fertile queens can remain hidden in shipments (Ward et al. 2006; Tschinkel 2013). We drew on historical Mediterranean data and recent occurrences from the Carpathian Basin involving four newly arrived Mediterranean species: *Camponotus lateralis* (Olivier, 1792), *Crematogaster schmidti* (Mayr, 1853), *Crematogaster scutellaris* (Olivier, 1792) and *Pheidole pallidula* (Nylander, 1849).

Our current study is—to our knowledge—the first to explore the potential association between rapidly changing regional climate and the northward dispersal of dominant ant species from the North Mediterranean territory. Our research is dedicated to providing in‐depth insights into the multifaceted challenges posed by climate change. We are confident that by investigating recent biological invasion trends at a local scale, we can contribute to a more comprehensive understanding of these environmental dynamics. By advancing our knowledge in this area, we can explore how local biological invasions are influenced by climate change and what implications this has for ecosystems and biodiversity as a whole, addressing the pressing issues associated with climate change.

## 2 Materials and Methods

### 2.1 Data Collection

As a first step, the occurrence data of non‐native ant species with a predominantly southern European distribution were collected from Hungary. We collected Hungarian distribution data of the four ant species ( *C. lateralis* , *C. schmidti* , *C. scutellaris* and *P. pallidula* ) of Mediterranean origin (see antmaps.org's species range maps, Janicki et al. 2016). It should be noted here that we have also included a record of *C. schmidti* from northern Serbia, which is located only nine kilometers from the Hungarian border and is a valuable addition to the Hungarian data. The majority of our new data from Hungary is gathered from the author's and citizen scientists' combined efforts, including social media postings and virtual and in‐person communication with participants. These data have been collected using citizen‐science methods from the “Hangyahatározó” Facebook group, which currently (09.10.2024) has 1607 amateur myrmecologist members, following the formerly tested approach (Báthori et al. 2022). The data were extracted from the date of the group's creation (19.03.2021) until 09.10.2024. The data uploaders were contacted and the following information was recorded about their findings: date, location, GPS coordinates, sex of the ants and voucher images of the specimens. The myrmecologist authors (SC and FB) confirmed the species identifications based on good quality photographs, or vouchers using Seifert (2018), hence, the labeled dataset of this project constitutes a reliable resource for subsequent statistical processing.

To determine historical “reference” distribution, reliable historical occurrence data for *C. lateralis* , *C. schmidti* and *C. scutellaris* prior to 2005 (“Mediterranean historical data set”) were extracted from the public databases available online at iNaturalist (https://www.inaturalist.org/), AntWeb (https://www.antweb.org) and AntWiki (https://www.antwiki.org). Since species‐level determination of *P. pallidula* is not possible based on images alone, we relied in this case on historical occurrence data published in Seifert (2016).

### 2.2 Statistical Analyses of Climatic Data

Meteorological data extraction and statistical analyses were performed in R 4.2.1 (R Core Team 2022). To establish reference years for climate comparison, we determined the median year of the Mediterranean historical occurrence records (1853–2004) and the median year of the Hungarian contemporary occurrence records (2019–2024). These median years (1982 and 2022, respectively) were used to define the time periods for extracting climatic data, enabling us to compare the climate of the Mediterranean region during the period when these ant species were widespread there (1980s) with the current environment of Hungary, where they are now establishing. Then, we obtained weather data from the E‐OBS daily gridded dataset v29.0e (Haylock et al. 2008; Cornes et al. 2018) at the ECAD website (European Climate Assessment and Dataset, http://www.ecad.eu), using the ‘ncdf4’ package (Pierce 2021) to access and extract NetCDF data files. In order to spatially match meteorological data to biological sampling sites, we identified the longitude‐latitude coordinates corresponding to each sampling site, and selected the 0.1° × 0.1° grid cell that contained the sampling site. From this grid cell, we used the apex that was geographically closest to the sampling location among those grid cell apices for which meteorological data were available to extract the corresponding meteorological data in the NetCDF file. If none of the apices of the relevant grid cell contained data in the NetCDF file, the sampling site was excluded from the analyses (see Table S2). As a result of this procedure, data could not be obtained for all Mediterranean locations (see Table S3). Hence, 66 Mediterranean and 15 Hungarian sites met the spatial and data‐availability criteria. We then removed duplicate grid coordinates as well as sites lacking complete data for all climatic variables. After these exclusions, the final dataset included 45 Mediterranean and 11 Hungarian locations (see Table S3). We extracted the following daily meteorological parameters: daily minimum temperature (°C), daily maximum temperature (°C), daily precipitation sum (mm), daily mean relative humidity (%), and daily solar radiation (Wm‐2). In order to minimize the influence of stochastic fluctuations in the climate, and since the most pronounced climatic changes have occurred during the last 20 years, we calculated each variable from the above raw weather parameters as the mean of two three‐year periods. Averaging data across multiple years is preferred over relying on a single year because it helps to smooth out short‐term anomalies (e.g., unusual cold years). Hence, using a 3‐year average can provide a more reliable and representative estimate of the typical climatic conditions for each period. Accordingly, for this we used the median years and the preceding two years, i.e., 1980–1982 and 2020–2022 (as during the analyses the data were not yet available in the E‐OBS database for the full year 2023), with the data for the years pooled within the historical and current periods to extract the following variables: mean minimum temperature (mean daily minimum temperature between 1 December and 31 March; in this case, the December preceding the year in question was included, as appropriate); mean maximum temperature (mean daily maximum temperature between 1 April and 30 September); mean precipitation (mean daily precipitation sum between 1 April and 30 September); mean humidity (mean of daily mean relative humidity between 1 April and 30 September); mean radiation (daily solar radiation between 1 January and 31 December). To capture climate conditions relevant to the establishment and survival of temperate‐zone ant species, we calculated seasonal climate variables: mean minimum temperature for the winter period (December–March), and mean maximum temperature, precipitation, and humidity for the vegetation period (April–September). Solar radiation was calculated for the entire year to capture annual energy availability. These periods were selected to represent key climatic constraints for temperate ectothermic species in general, not species‐specific requirements. Because of the different overwintering and nesting sites of ant species and the temperature conditions they experience (Mitrus 2013), solar radiation was investigated for the whole year. The derived meteorological data are presented in Table S3.

Our aim was to investigate whether there is a difference in weather variables between historical and contemporary Mediterranean and Hungarian habitats. Specifically, we focused on two key comparisons: (1) historical Mediterranean vs. historical Hungarian climate to establish the baseline climatic difference that existed in the 1980s, and (2) historical Mediterranean vs. contemporary Hungarian climate to test whether this climatic barrier has diminished or disappeared due to recent warming. Since the variances of the groups were highly different (Levene's tests, all *p* < 0.01), we used the Kruskal‐Wallis test to compare the groups, applying the kruskal.test() function, and dunn.test() function of the ‘dunn.test’ package (Dinno and Dinno 2017) for pairwise comparisons, applying the Benjamini and Hochberg (1995) correction for false discovery rate.

### 2.3 Figure Production

The map representing the distribution data was created with QGIS version 3.10.6 (QGIS Development Team 2020) and used NASA's SRTM elevation data (urs.earthdata.nasa.gov) and the DIVA‐GIS country administrative area data (https://diva‐gis.org/index.html) to display the country boundaries. The montage of habitat photos and the addition of country names to the map was done using the free PhotoScape ver. 3.7.

## 3 Results

### 3.1 New Ant Species Occurrences in Hungary

**FIGURE 1:**
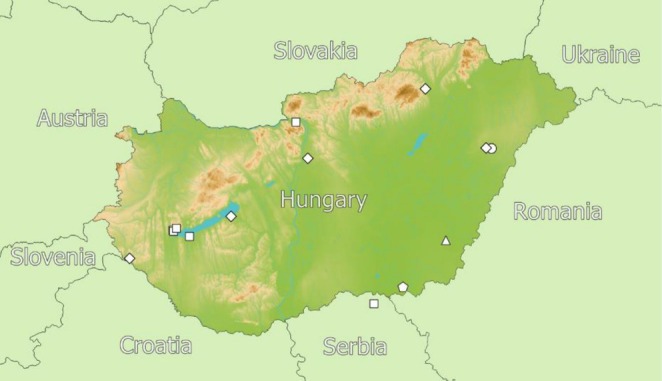
Recently reported distribution data include *Camponotus lateralis* (circle), *Crematogaster schmidti* (square), *Crematogaster scutellaris* (diamond), *Pheidole pallidula* (pentagon), and an unidentified *Pheidole* sp. (triangle) ant species from Hungary and Serbia.

Based on the data uploaded to the “Hangyahatározó” Facebook group, seven previously unknown and unpublished occurrences of *C. schmidti* , five occurrences of *C. scutellaris* , one occurrence of *C. lateralis* , one occurrence of *P. pallidula* , and one occurrence of *Pheidole* sp. from the Carpathian Basin have been reported (see Figure 1 and Table S1).

**FIGURE 2:**
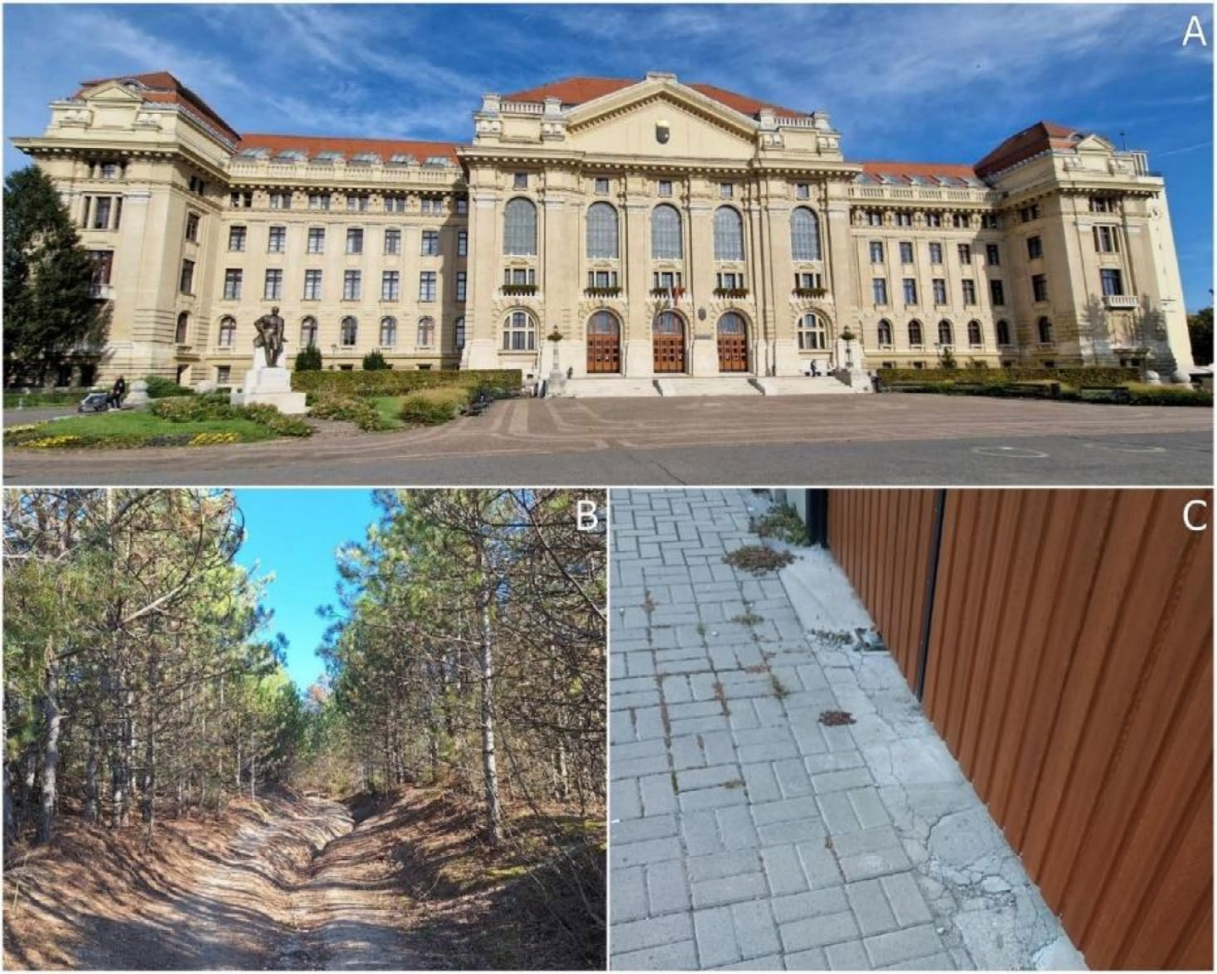
A highly urbanized, paved square in Debrecen, where *C. lateralis* and *C. scutellaris* are found co‐occurring (A) (photo: Ferenc Báthori). Pine forest near Gyenesdiás, habitat where *C. schmidti* occurs (B) (photo: Tamás Jégh). Paved sidewalk in Makó, where *P. pallidula* is nesting among the paving stones on the street front of a residential building (C) (photo: Jenő Kiss).

Of the five observations of *C. scutellaris* (all observations from different data providers), three were from mature colonies, in one case (near Csörnyeföld) a single alate and in another (Budapest) a single worker was observed in the field. These data for *C. scutellaris* were all from highly urbanized, inner‐city environments (e.g., Figure 2A). In the case of *C. schmidti* , all observations come from adult colonies (from a total of seven different observers), and in some locations the species has been present for several years (e.g., in Keszthely since 2019). These data were typically from parts of urbanized habitats that were characterized by green surfaces (parks, gardens, planted forests), in most cases with southern exposure (Figure 2B). One adult colony of *P. pallidula* was observed in the highly urbanized inner area of Makó from the street frontage of a garden house (Figure 2C), while an alate was observed in the inner area of Békéscsaba. The only record (a single colony) of *C. lateralis* was found in a square of a highly urbanized area of Debrecen (Figure 2A), where it is found in parabiosis with *C. scutellaris* from 2022 (still present in autumn 2024, based on FB's personal observation).

### 3.2 Climatic Shifts

**FIGURE 3:**
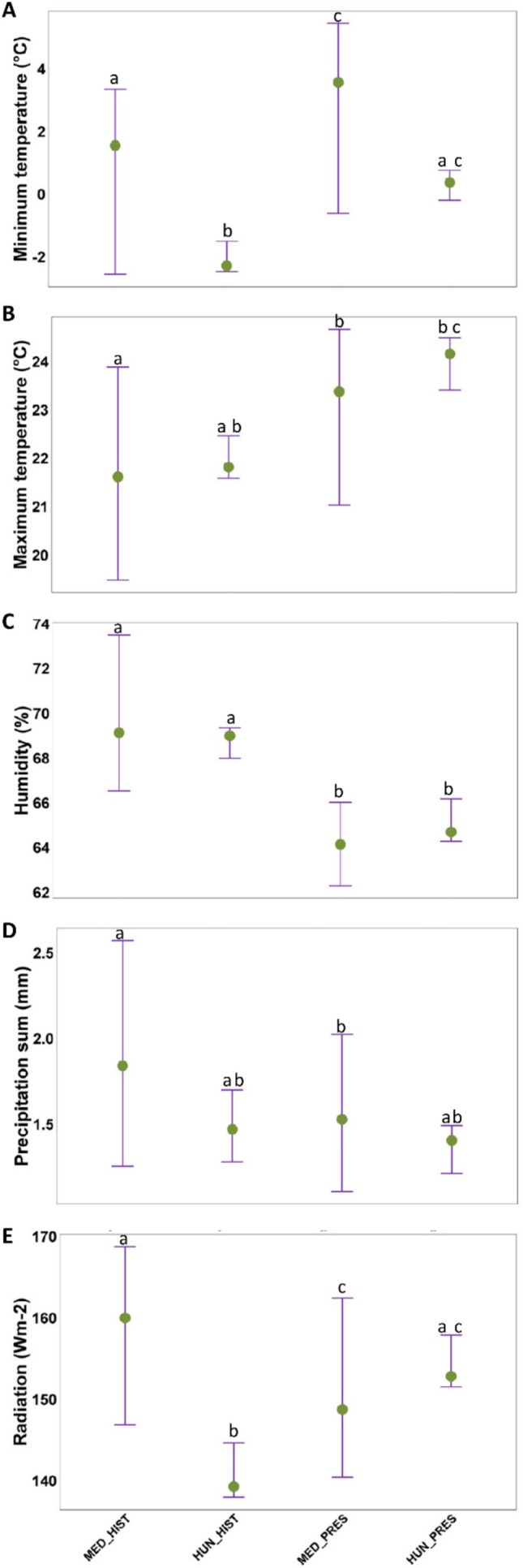
Comparisons of meteorological variables (median ± IQR) between the historical and contemporary Mediterranean and Hungarian region [(A) Minimum temperature (°C), (B) Maximum temperature (°C), (C) Humidity (%), (D) Precipitation sum (mm), (E) Radiation (Wm‐2)]. Letter codes (a–d) indicate the statistically significant (*p* < 0.05) differences between groups, as revealed by post hoc comparisons. Abbreviations: HUN_HIST, Hungarian historical; HUN_PRES, Hungarian present; MED_HIST, Mediterranean historical; MED_PRES, Mediterranean present.

We found differences between groups in all derived meteorological variables (mean minimum temperature: *χ* ^2^ = 16.52, df = 3, *p* < 0.001; mean maximum temperature: *χ* ^2^ = 12.28, df = 3, *p* = 0.007; mean precipitation: *χ* ^2^ = 8.76, df = 3, *p* = 0.03; mean humidity: *χ* ^2^ = 32.71, df = 3, *p* < 0.001; mean radiation: *χ* ^2^ = 20.24, df = 3, *p* < 0.001). All pairwise comparisons are summarized in Table 1 and Figure 3. Within areas, the historical—contemporary changes were consistent with climate change predictions, i.e., the climate became hotter and drier. In the historical Mediterranean vs. historical Hungarian comparison, we found lower mean minimum temperature and radiation in Hungary, but this difference disappeared in the historical Mediterranean vs. contemporary Hungarian comparison. Further, maximum temperature was higher and humidity lower in the contemporary Hungarian than in the historical Mediterranean locations. Interestingly, precipitation did not differ in any Mediterranean—Hungarian comparisons. Collectively, climate change made contemporary Hungarian locations climatically comparable to (or even drier and hotter than) the historical Mediterranean locations, while the Mediterranean locations themselves became much drier and hotter in the studied period.

**TABLE 1:** Adjusted *p*‐values (after Benjamini‐Hochberg correction) of pairwise comparisons of meteorological variables between the Hungarian (Hun) and Mediterranean (Med) area from historical (1980–1982; HIST) and present (2020–2022; PRES) periods.

	Med_HIST vs. Med_PRES	Med_HIST vs. Hun_HIST	Med_HIST vs. Hun_PRES	Med_PRES vs. Hun_HIST	Med_PRES vs. Hun_PRES	Hun_HIST vs. Hun_PRES
Minimum temperature	0.02	0.02	0.41	0.0003	0.060	0.016
Maximum temperature	0.02	0.49	0.012	0.084	0.12	0.023
Precipitation	0.02	0.20	0.089	0.40	0.43	0.48
Humidity	< 0.0001	0.40	0.007	0.0007	0.34	0.015
Radiation	0.007	< 0.0001	0.25	0.008	0.19	0.007

## 4 Discussion

The results of our study are consistent with the findings that during the last decades, Hungary's climate changed dramatically (e.g., Bartholy and Pongrácz 2007; Breuer et al. 2017), increasingly mirroring some aspects of the Mediterranean climate of the 1980s. Our results suggest that climatic conditions in the new Hungarian colonization sites of these ant species today (2020–2023) have become similar to the climatic conditions in their historical (1980–1982) Mediterranean distribution area. More specifically, in the historic period, the minimum temperature and the radiation differed between the two regions, but these two parameters have become similar between the historical Mediterranean and contemporary Hungarian areas, eliminating the climatic barrier that previously might have prevented these Mediterranean ant species from establishing viable populations in Hungary. Mediterranean species can additionally benefit from elevated temperatures, as they may be better suited to these conditions than indigenous species, remaining active during times when native species cannot (Destour et al. 2024). Additionally, ecological naivety in the absence of co‐evolutionary history can further reduce biotic resistance and facilitate invasion success. These taxa could take advantage of this opportunity, given the current climate change, and expand their range into new northern territories, exploring previously uncharted areas and reshaping local ecosystems.

Although several ant species can dynamically respond to changes in microclimatic factors in their habitats by changing the location of their nests (McGlynn 2012; Sankovitz and Purcell 2021), on a larger scale, climatic conditions can still have a decisive influence on their distribution (Nascimento et al. 2022; Parr and Bishop 2022). One such critical factor that may influence the distribution of species with a predominantly Mediterranean range is the winter conditions (Battisti et al. 2005; Szyniszewska et al. 2024). Our results show that in the studied historical period (between 1980 and 1982) the Hungarian areas had significantly lower minimum winter temperatures than the Mediterranean area, but this difference vanished after 40 years of climate change. Combined with the found increased solar radiation and the fact that summers in the Carpathian Basin are becoming hotter and drier in line with Bartholy et al. (2009) and Rakonczai (2011), as is characteristic of the Mediterranean (Lionello et al. 2006), the climatic barriers that once restricted these species to southern regions have effectively disappeared, enabling their northward expansion.

The increasing frequency of human‐facilitated species introductions, coupled with the rapid changes in climate, creates a perilous situation for ecosystems on both local and regional scales (Seebens et al. 2017; Schlaepfer and Lawler 2023). As climate conditions shift, many organisms can now thrive in new habitats that were previously deemed unsuitable or even inaccessible just a few decades ago. This phenomenon is particularly evident in a growing body of research focusing on non‐native species, which highlights how these organisms are able to expand their ranges and colonize areas that are becoming more conducive to their survival. Studies conducted by researchers such as Hsiang et al. (2017), Borrelli et al. (2020), Kalkuhl and Wenz (2020), and Mormul et al. (2022) provide compelling evidence of this trend, illustrating the potential for invasive species to disrupt local ecosystems and outcompete native flora and fauna.

The northward spread of temperate species is influenced by two main factors, increased transport activity (Éltető and Völgyi 2013) and the effects of climate change on habitat suitability and species distribution (Harrison et al. 2006; Langham et al. 2015; Nuttall 2022). Transport activity—albeit to a lesser extent—has existed before, allowing species to spread beyond the natural scale. However, as demonstrated by our climatic analysis, the regional climate has now converged with historical Mediterranean conditions, providing the opportunity for establishing sustainable populations that would not have been viable four decades ago. The Hungarian imports alone increased 1.5–2 times from the early 2000s to the early 2010s from almost all regions (Éltető and Völgyi 2013), significantly increasing the potential for introduction of non‐native species in this region. The risk is further exacerbated by the more intensive movement of people and goods and the heat island effect of cities (i.e., a phenomenon where urban areas experience higher temperatures than their rural surroundings; Deilami et al. 2018), which has been shown to contribute to the establishment of non‐native species in urban areas (Király et al. 2014; Francis and Chadwick 2015; Báthori et al. 2024; López‐Collar et al. 2024; Destour et al. 2025). Given the fact that despite ongoing efforts to meet climate protection goals yield limited success and the chances of breaking the trend of global average temperature rise are remote (Cass 2012; Brown et al. 2019; Maslin et al. 2023), the need for improving imported transport quarantines is an immediate, demanding action.

The emergence of studied ant species with a predominantly Mediterranean distribution in the Carpathian Basin was to be expected, since all of the ant species studied are found over a relatively large area in the countries neighboring Hungary (Seifert 2018), mainly to the south and west, such as Austria (Steiner et al. 2017), Slovenia (Bračko 2007), Croatia (Bračko 2006), Serbia (Petrov and Collingwood 1992), and Romania (Markó et al. 2006), but almost all of the newly discovered occurrences can be considered as the northernmost occurrences in the Carpathian Basin. In contrast, *C. scutellaris* , which has a mainly western Mediterranean distribution (Seifert 2018), does not seem to be able to follow *C. schmidti* through these habitats according to recent data, but has been introduced in many urbanized habitats (mainly in cities) through human mediation (Boer and Vierbergen 2008; Seifert 2018). This species has been supposed native to Hungary based on previous literature (Mayr 1861; Csősz et al. 2021) without evidence. However, we believe that this Mediterranean species may have been introduced, as it currently exists only in urbanized areas, often within city centers (with probably more favorable microclimate), suggesting that human activities likely led to its accidental introduction, though the recent convergence of Hungarian climate with Mediterranean conditions likely facilitates the species' ability to establish viable populations in Hungary. This seems to be supported by the fact that in recent years the species has appeared in isolated patches in distant cities, relatively far from natural habitats, usually in areas with strong human influence and typically warm microclimatic conditions. The present occurrence of *C. lateralis* in Hungary is probably the result of such an accidental introduction, since the only known occurrence so far is the marbled, paved area of the main square of the university in Debrecen, where it co‐occurs with *C. scutellaris* . It is known that these two species often live in parabiosis with each other (Menzel et al. 2010; Seifert 2018), so considering that despite relatively frequent ant surveys in the city (by András Tartally and Ferenc Báthori), *C. lateralis* and *C. scutellaris* have not been detected in the area in the last two decades until 2022, a single co‐introduction event is likely at this location in the recent past. Human introduction is also likely in the two *Pheidole* records found in the south‐eastern part of the country (near Makó and Békéscsaba), where the species was found in urbanized environments. However, these southern areas are so close to the known native ranges of the species that we cannot exclude a possible natural northward expansion of the species, especially as one of the distribution records is from a freshly swarmed winged alate individual. While the arrival of alien species generally has detrimental effects on local ecosystems, we should not overlook that these non‐natives can also have positive ecological and socioeconomic impacts, although these are rarely documented (Vimercati et al. 2020). For example, it is known that native plants can be highly dependent on seed dispersal by invasive birds (Vizentin‐Bugoni et al. 2019).

In summary, Hungary is experiencing substantial and multi‐faceted impacts of climate change that require urgent attention and action. These influences align with the climate trends observed in Mediterranean regions in the last decades, threatening local ecosystems. As Hungary's climate continues to warm and change, it is becoming increasingly hospitable for Mediterranean species to establish themselves within its borders. This process is further facilitated by the extending trading network and increasing transport activities. The transforming climate poses a significant challenge, as these newly introduced species can disrupt local biodiversity, outcompete native flora and fauna, and alter existing habitats in ways that are difficult to reverse. Since current climate change mitigation strategies do not guarantee quick success, we advocate for the establishment of effective (even regional) control strategies to prevent the human‐mediated introduction of non‐native species. By investing in research, monitoring activity, and public awareness campaigns, we can both better understand the dynamics of these changes and formulate strategies that protect Hungary's environmental integrity.

## Author Contributions

**Sándor Csősz:** conceptualization (equal), methodology (equal), writing – original draft (equal). **Miklós Laczi:** conceptualization (equal), data curation (equal), formal analysis (equal), methodology (equal), software (equal), writing – original draft (equal). **Gábor Herczeg:** conceptualization (equal), methodology (equal), writing – original draft (equal). **Ferenc Báthori:** conceptualization (equal), data curation (equal), methodology (equal), software (equal), writing – original draft (equal).

## Funding

This work was supported by the National Research, Development and Innovation Office (Hungary, #K‐147781) and the HUN‐REN Hungarian Research Network.

## Conflicts of Interest

The authors declare no conflicts of interest.

## Supporting information


**Table S1:** New occurrences of the studied species from Hungary and Serbia.

**Table S2:** The geographical coordinates of the specimens and the coordinates assigned from the E‐OBS database.

**Table S3:** The derived meteorological data of the locations.

## Data Availability

Data available in article Supporting Information.
